# Treatment of Feline Lung–Digit Syndrome with Toceranib Phosphate: Prolonged Survival and Novel Metastatic Findings

**DOI:** 10.3390/ani16050839

**Published:** 2026-03-07

**Authors:** Inês Cabral, Gustavo Matos, Gabriela Fernandes Silva, Fátima Carvalho, Irina Amorim

**Affiliations:** 1 ICBAS-School of Medicine and Biomedical Sciences, Porto University, Rua de Jorge Viterbo Ferreira 228, 4050-313 Porto, Portugal; 2 Egas Moniz Centre for Interdisciplinary Research (CiiEM), Egas Moniz School of Health & Science, 2829-511 Almada, Portugal; 3 Institute of Molecular Pathology and Immunology, University of Porto (IPATIMUP), Rua Júlio Amaral de Carvalho Nr. 45, 4200-804 Porto, Portugal; 4 Institute for Research and Innovation in Health (i3S), University of Porto, 4200-135 Porto, Portugal

**Keywords:** toceranib phosphate, pulmonary carcinoma, feline lung–digit syndrome, MODAL syndrome, c-kit, immunohistochemistry

## Abstract

Feline pulmonary carcinomas are rare and often carry a poor prognosis, particularly when associated with feline lung–digit syndrome. We report a cat with primary pulmonary carcinoma and extensive metastases—including digits, pleura, mandible, scapula, spleen, skeletal muscle, and distant lymph nodes—supporting the broader “MODAL syndrome” concept. Palliative therapy with toceranib phosphate and meloxicam achieved prolonged survival and excellent quality of life, with no adverse effects despite dose escalation. Rapid progression after discontinuation suggests a role for toceranib in delaying tumour growth. Immunohistochemistry revealed c-kit expression in one metastatic lesion but not in the primary tumour or most metastases, highlighting intratumoral heterogeneity and the complexity of targeted therapy. The observed benefit likely reflects toceranib’s multi-target activity (VEGFR2, PDGFR), impacting angiogenesis and tumour progression. This case represents the first report of toceranib phosphate use in feline pulmonary carcinoma and underscores its potential as a palliative option.

## Simple Summary

Feline lung–digit syndrome is a metastatic pattern in which a primary pulmonary carcinoma spreads to the digits, often leading to delayed diagnosis and poor prognosis. Currently, no standard effective treatment exists. In this case, a cat with extensive metastatic disease was treated with toceranib phosphate as palliative therapy, achieving survival well beyond the average reported for surgical intervention alone and maintaining excellent quality of life. Post-mortem analysis revealed widespread metastases and c-kit expression, a molecular target of toceranib, in one lesion, highlighting tumour heterogeneity and the complexity of targeted therapy. This is the first report of toceranib use in feline pulmonary carcinoma, suggesting its potential as a palliative option.

## 1. Introduction

Feline lung–digit syndrome (FLDS) is rare and refers to an unusual metastatic pattern from a primary pulmonary carcinoma into one or more digits, which can possibly affect multiple limbs [1]. Recently, the replacement of FLDS with MODAL (muscle/ocular/digit/aorta/lung) syndrome has been suggested, as a reminder that other sites of metastasis frequently occur [2]. Additional reported locations for metastases are the pleural cavity, bronchial lymph nodes, skin, liver, spleen, heart, brain, kidney, eye, intestine, bone, omentum and adrenal gland [1].

Primary pulmonary tumours might be clinically silent, and patients might only show clinical signs related to metastases and their location when the disease has often progressed considerably [2]. Consequently, the late presence of clinical signs and delayed diagnosis leads to a short mean survival time of 58 days, ranging from 12 to 122 days [3]. Treatment for this disease has proved to be difficult due to its rapid progression [2].

Toceranib phosphate (TOC) is a tyrosine kinase inhibitor that was first approved as a therapy for mast cell tumours in dogs, but that has proved to be useful in canines against other neoplasms, such as carcinomas [4]. It blocks different surface receptors in the tumoral cells, such as c-kit, vascular endothelial growth factor receptor 2 (VEGFR2), platelet derived growth factor receptor (PDGFR), and Feline McDonough Sarcoma tyrosine kinase 3 [4,5]. These receptors, when activated or overexpressed in neoplastic cells, promote tumour growth and metastatic dissemination [6].

This pharmaceutical agent has been used off-label in cats with different types of carcinomas and appears to be well tolerated [5,7,8,9,10,11,12]. The major side effects reported are uncommon and can include gastrointestinal upset (21.8%), thrombocytopenia (16.3%), azotaemia (14.5%), neutropenia (9.1%) and alanine aminotransferase elevations (7.2%) [13].

This case report describes a cat that was treated with palliative TOC after being diagnosed with FLDS.

## 2. Case Presentation

A 12-year-old, domestic long-haired, indoor, neutered female cat was presented to the veterinary clinic with forelimb lameness graded 7 out of 10, accompanied by swelling of the fourth distal digit on the left forelimb. An additional nodular lesion was found on the fifth digit of the same limb. A seven-day course of oral meloxicam at a dosage of 0.1 mg/kg every 24 h was initiated. One week following initial presentation, ulceration of the fourth digit was observed, prompting culture and sensitivity testing, which identified an *Escherichia coli* infection. Oral co-amoxiclav (Penamoxvet^®^ 25 mg/mL + 6.25 mg/mL) was subsequently administered at a dosage of 20.0 mg/kg. No clinical improvement was noted after seven days of antimicrobial therapy.

A complete blood count, serum biochemistry, and digital and thoracic radiographs were performed. Both the haematological and biochemical parameters were within normal limits. Radiographic examination of the digits revealed osteolysis of the third phalanx of the fourth digit without evidence of intra-articular invasion, as well as another lesion affecting the fifth digit. Right lateral, left lateral, and dorsoventral thoracic radiographs demonstrated a solitary, well-circumscribed mass located in the caudal pulmonary lobe. A round, air-filled cavitary lesion surrounded by soft tissue was also observed. No pleural effusion or enlargement of the tracheobronchial lymph nodes was noted.

Thoracic auscultation revealed no abnormalities, and there were no clinical signs of dyspnoea, tachypnoea, or increased respiratory effort. At the time, the cat was clinically stable and in good general condition.

A partial amputation of the fourth digit was performed due to the 2.0 cm ulcerative mass. In addition, the lesion on the fifth digit, measuring 1.1 cm, was excised. Both tissue samples were submitted for histopathological evaluation in a third-party laboratory, revealing a poorly differentiated squamous cell carcinoma on the fourth digit and an infundibular follicular cyst on the fifth digit. A presumptive diagnosis of FLDS was made.

The cat recovered well from surgery, with no post-operative complications observed. During the post-operative recovery period, a new round lesion was detected on the lateral side of the left mandible, measuring about 1.7 cm in diameter, and suspected to represent an osseous metastatic lesion. Despite this finding, the cat remained clinically well, with no reduction in appetite or other clinical signs.

The poor prognosis was discussed with the owners, along with potential palliative treatment options, including carboplatin and TOC. Due to concerns regarding potential adverse effects, TOC was initiated at a dose of 2.3 mg/kg administered every Monday, Wednesday and Friday. After two weeks of treatment, the dosage was increased to 3.1 mg/kg. The cat was concurrently receiving meloxicam at 0.1 mg/kg once daily, with no side effects reported. The owner was provided with a “Pet Quality of Life Scale and Daily Diary” and throughout TOC treatment, the cat’s quality of life was assessed with a mean score of 10.5 out of 12 [14].

A complete haematology and serum biochemistry profile were performed prior to the first administration of TOC, two weeks after initiating treatment, and subsequently monthly. No evidence of myelosuppression or elevations in hepatic or renal parameters were observed, and no gastrointestinal adverse effects were reported. For analgesia, buprenorphine was administered transmucosally at a dose of 0.03 mg/kg every 6 h. Time to progression was defined as the interval from the first TOC administration until radiographic and clinical evidence of disease progression, based on serial measurements of the mandibular mass made with a calliper. Progression was documented at 65 days, at which point TOC treatment was discontinued. Following this, rapid disease progression was observed, not only in the mandibular lesion but also with enlargement of the right popliteal lymph node and the appearance of additional nodular lesions, the most prominent located in the right scapular region, so the RECIST criteria were applied to consider it progressive disease. Severe weight loss was also noted during the progression of the disease and, in addition due to it being very difficult to administer the toceranib tablets to the cat, it was decided to discontinue the cytostatic treatment. Even though toceranib was no longer being administered, the owner reported that the cat’s quality of life was appropriate and analgesia was still provided by meloxicam 0.1 mg/kg orally every 24 h and buprenorphine transmucosally at a dose of 0.03 mg/kg every 6 h. The use of gabapentin was also discussed at 7 mg/kg orally every 8 h. Due to the rapid advancement of the disease condition and the resulting compromise in quality of life, including analgesia management, euthanasia was elected. Survival time was calculated from the initial clinical presentation until the time of death, totalling 122 days.

## 3. Materials and Methods

### 3.1. Necropsy and Histopathological Evaluation

Written consent for necropsy was obtained from the owners, and the cadaver was submitted to the Veterinary Pathology Laboratory of the School of Medicine and Biomedical Sciences (ICBAS), University of Porto, for post-mortem examination. A full necropsy protocol was performed to assess the extent of the neoplastic condition. Tissue samples were collected from all identified lesions, which were systematically documented and measured. In cases where no macroscopic abnormalities were observed, representative samples of all major organs were nonetheless collected for histopathological evaluation, ensuring a comprehensive microscopic assessment.

The collected samples were submerged and fixed in 10% neutral buffered formalin, routinely processed, embedded in paraffin wax, sectioned at 2 μm thickness, and stained with haematoxylin and eosin (H&E) for microscopic evaluation. Additionally, unstained histological sections from the first identified digital lesion were kindly provided by the external laboratory, enabling reassessment, inclusion and comprehensive evaluation of all lesions, as well as comparative molecular analyses between lesions before and after the instituted treatment.

### 3.2. Immunohistochemical Study

With the aim of confirming the histogenesis of the lesions, evaluating the expression of therapeutic targets relevant to the treatment administered, and gaining further insight into the biological behaviour and progression of the neoplastic disease, a targeted immunohistochemical panel was performed on selected samples considered representative and diagnostically relevant. Antigen retrieval was carried out using a Target Retrieval Solution (Dako, Santa Clara, CA, USA) and, for visualization, the Novolink^TM^ Max-Polymer detection system (Novocastra, Newcastle, UK) was used, in accordance with the manufacturer’s instructions. The slides were incubated in a humid chamber overnight at 4 °C, with anti-pancytokeratin (clone AE1/AE3, Invitrogen, Carlsbad, CA, USA; dilution factor 1:300), anti-thyroid transcription factor-1 (TTF-1) (clone IHC141, GenomeMe, Richmond, BC, Canada; ready to use) and anti-c-kit (clone CD117, Dako, Santa Clara, CA, USA; dilution factor 1:450). Between each step of the procedure, sections were washed with triphosphate-buffered saline. 3,3-diamino-benzidine (DAB) (Sigma, St. Louis, MO, USA) was then used as the chromogen, with haematoxylin serving as counterstaining. For negative controls, the primary antibody was replaced with another of the same immunoglobulin isotype. Positive controls consisted of canine tissue sections of normal uterine tissue, normal thyroid and a cutaneous mast cell tumour, known to express AE1/AE3, TTF-1 and c-kit, respectively.

## 4. Results

### 4.1. Necropsy

**Figure 1:**
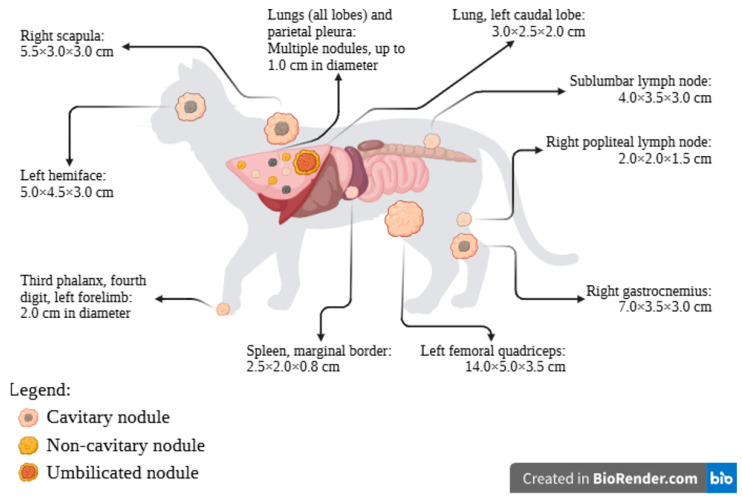
Anatomical distribution and gross features of metastatic masses identified at necropsy. Despite the variability in size, all lesions were irregular, multinodular and presented poorly defined margins. Created in BioRender.com, accessed on 21 November 2025.

**Figure 2:**
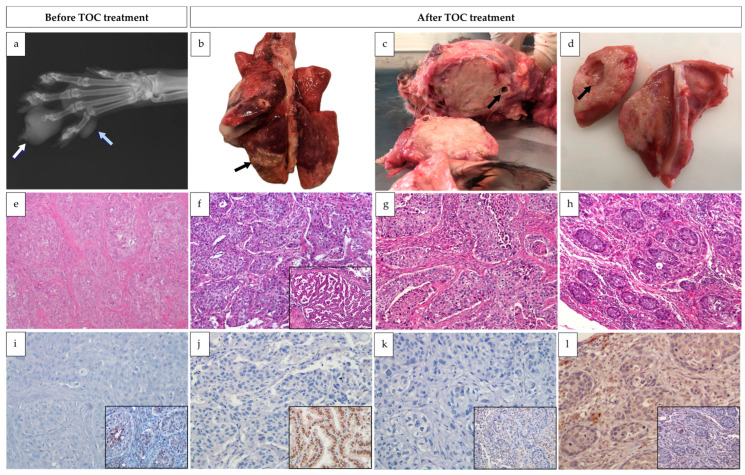
Main lesions and histopathological and immunohistochemical investigations, before and after treatment with toceranib phosphate. (**a**) Radiography of the left forelimb, dorso-palmar projection. An extensive mass can be identified, causing osteolysis of the third phalanx of the fourth digit (white arrow). Furthermore, a smaller nodule can be discerned in the fifth digit (blue arrow). (**b**) Cardiorespiratory system. A larger, yellow, umbilicated lesion is present in the left caudal lobe (arrow) and there are smaller white, yellow or grey masses, multifocally and randomly distributed in all lobes. (**c**) Sagittal cuts of the head. An extensive mass occupies the left side of the head, extending from the ocular region into the ear canal (arrow). (**d**) Right scapula. Infiltrating the right scapular bone and surrounding skeletal muscles, there is an extensive white, multinodular, irregularly shaped mass, with poorly defined borders and a central cavity (arrow). (**e**) Fourth digit lesion in the left front limb. There are nests of polygonal cells, surrounded by moderate amounts of fibrous tissue (H&E, 100×) (**f**) Lung, squamous portion of the neoplasm. Alveolar spaces are invaded by nests of polygonal neoplastic cells (H&E, 100×). Inset: Arborizing fronds of columnar cells, supported by scant fibrovascular stroma, form the papillary portion of the neoplasm (H&E, 100×). (**g**) Left hemiface lesion. The presence of neoplastic cells triggers a noticeable desmoplastic reaction (H&E, 100×). (**h**) Right scapular lesion. Multifocally throughout the tissue, there is formation of round to oval structures containing neoplastic cells (H&E, 100×). (**i**) Fourth digit mass. There is no immunoreactivity against c-kit (200×). Inset: About 10% of neoplastic cells have moderate nuclear markings against TTF-1 (200×). (**j**) Primary lesion in the lung. None of the cells show immunopositivity against c-kit (200×). Inset: The majority of the cells that form the papillary portion of the tumour have marked nuclear and paranuclear immunoreactivity against TTF-1 (400×). (**k**) Metastatic hemifacial lesion. C-kit cannot be detected using immunohistochemistry. (200×). Inset: A few cells show moderate nuclear immunoreactivity against TTF1 (200×). (**l**) Right scapular lesion. A faint, diffuse cytoplasmatic immunoreactivity against c-kit can be seen in the majority of neoplastic cells (200×). Inset: There is rare immunopositivity against TTF-1 in the nuclei of neoplastic cells (200×).

At necropsy, the animal exhibited poor body condition, with evident signs of cachexia, including marked loss of adipose tissue and skeletal muscles. Necropsy examination confirmed the previously reported masses and revealed additional metastatic lesions, compatible with advanced metastatic disease. A comprehensive list of all identified masses and their anatomical locations is provided in Figure 1 and the radiograph of the lesion on the fourth digit, previously excised, is presented in Figure 2a.

The lesion previously identified through imaging in the caudal left pulmonary lobe measured 3.0 × 2.5 × 2.0 cm. It was white to yellow in colour, umbilicated, elevated, and exhibited poorly defined margins (Figure 2b). All pulmonary lobes contained randomly distributed white, grey or yellow nodules up to 1.0 cm in diameter (Figure 2b). Another prominent lesion included a mass located in the left hemiface, extending from the oral cavity and involving the parotid area and the orbit. It measured approximately 5.0 × 4.5 × 3.0 cm, consisted of firm white tissue, and contained a central area of purulent material (Figure 2c), involving both bone and skeletal muscles. Additionally, masses located within the right popliteal lymph node as well as in the right scapular bone and adjacent muscles were also confirmed (Figure 2d). Other masses, of variable size and with morphological characteristics similar to those previously described, were identified in several locations, including the spleen, sublumbar lymph node, left femoral quadriceps and right gastrocnemius muscles (Figure 1).

### 4.2. Histopathology

Microscopically, the pulmonary mass consisted of a population of neoplastic epithelial cells displaying two distinct patterns. The predominant pattern, constituting 80% of the neoplasm, comprised polygonal cells arranged in sheets and nests, supported by scant fibrovascular stroma and infiltrating alveolar spaces. These cells exhibited granular to fibrillar eosinophilic or occasionally optically empty cytoplasm, variably defined borders, and round to oval nuclei with stippled chromatin. There was moderate anysokaryosis and marked anisocytosis (Figure 2f). The second pattern, representing 20% of the lesion, consisted of papillae lined by simple columnar epithelium supported by scant fibrovascular stroma. These epithelial cells had eosinophilic cytoplasm with well-defined borders, frequently bore cilia, and contained a basal oval nucleus with stippled chromatin and, occasionally, a prominent nucleolus (Figure 2f—inset). The mitotic count was 11 per 2.37 mm^2^, with occasional bizarre mitoses. Multifocally, moderate areas of necrosis were present, associated with dystrophic calcification and cholesterol cleft formation, as well as mild peritumoral lymphoplasmacytic infiltrates. This lesion was diagnosed as a pulmonary adenosquamous carcinoma.

The other masses exhibited similar microscopic characteristics. In general, there was disruption of the normal tissue architecture, which was replaced with round to oval structures or sheets of neoplastic epithelial and polygonal cells (Figure 2e,g,h), showing marked nuclear pleomorphism and frequent mitotic figures. Moderate to marked proliferation of fibrous tissue accompanied the presence of these neoplastic cells. A presumptive diagnosis of a primary pulmonary adenosquamous carcinoma, with metastasis to the lung, parietal pleura, left hemiface, right scapula, spleen, popliteal and sublumbar lymph nodes, left femoral quadriceps and right gastrocnemius, was considered.

### 4.3. Immunohistochemistry

The immunohistochemical panel was performed on slides representative of the first biopsy taken from the fourth digit of the left forelimb, as well as on lesions subsequently identified during necropsy, located in the lung, scapular region and left hemiface.

In all the lesions tested, the great majority of the neoplastic population (>95%) showed moderate-to-strong AE1/AE3 immunopositivity, both membranous and cytoplasmatic, confirming the epithelial histogenesis of the lesions.

In addition, approximately 90% of the neoplastic cells constituting the papillary portion of the pulmonary lesion exhibited moderate nuclear to paranuclear TTF-1 immunoreactivity (Figure 2j—inset). In the squamous counterpart, only about 5% of the cells exhibited mild-to-moderate nuclear TTF-1 immunopositivity. In all other lesions, 0–10% of the neoplastic cells displayed weak to moderate nuclear positivity against the same antibody (Figure 2i—inset, Figure 2k—inset, Figure 2l—inset).

Regarding c-kit immunoexpression, no immunoreactivity was observed in the neoplastic cells present in the digit, lung, or facial lesion. (Figure 2i–k). However, approximately 90% of the neoplastic cells in the scapular lesion exhibited weak cytoplasmic c-kit immunopositivity (Figure 2l).

The immunopositivity against TTF-1 in the pulmonary lesion made it possible to diagnose carcinoma of pulmonary origin. Although more uncommon, the presence of this antigenic marking in other lesions permitted connecting them to the one found in the lungs, classifying them as metastases arising from a primary pulmonary carcinoma.

## 5. Discussion

Pulmonary carcinomas in cats are rare and have a poor prognosis; therefore, there are limited data regarding treatment options and response [2]. Amputation of the affected digit can be recommended as a palliative measure to enhance the cat’s comfort and overall quality of life. Indeed, Gottfried et al. (2000) compiled clinicopathological findings from a group of cats with metastatic digital carcinoma originating from primary pulmonary carcinoma [3]. Among the 19 cases with complete clinical records, the median survival time from onset of clinical signs was only 58 days [3]. Digit amputation was occasionally performed as a palliative measure, but disease progression, often involving additional digits and systemic decline, was common, limiting its benefit and with survival typically less than two months after diagnosis [3].

Regarding treatment, one study reported pneumonectomy performed in a cat with a well-differentiated adenocarcinoma of pulmonary origin, with posterior mitoxantrone chemotherapy resulting in survival of the animal for at least 34 months, but there was no evidence of metastatic disease [15]. The usage of tyrosine receptor inhibitors has been suggested as a possible palliative treatment for this disease, as it has been successfully used for other feline carcinomas, but it has never been specifically reported in FLDS [2]. A recent study on cats with oral squamous cell carcinoma treated with TOC reported that patients responding to this therapy experienced longer progression-free survival and overall survival times [5].

Recently, a case report described a dog with primary pulmonary adenocarcinoma that developed pulmonary metastases three months after lobectomy [16]. TOC therapy was initiated at month ten and continued long-term, with dose adjustments due to adverse effects such as gastrointestinal signs and neutropenia [16]. The dog survived for 33 months post-diagnosis, suggesting that TOC may offer a valuable therapeutic option for metastatic pulmonary adenocarcinoma [16].

Similarly, the cat of the present report showed a longer survival time than the mean previously described [3]. Additionally, the disease seemed to progress rapidly after stopping TOC administration, suggesting that it may have held off the progression of the disease during its intake.

The owner was provided with a “Pet Quality of Life Scale and Daily Diary” to monitor the cat’s well-being throughout TOC treatment. This instrument evaluates key parameters such as appetite, mobility, social interaction, and overall comfort, offering a structured approach to assess daily quality of life. During therapy, the cat achieved a mean score of 10.5 out of 12, indicating a very good level of comfort despite the underlying disease and ongoing treatment. Notably, no adverse effects were observed during treatment, and the cat appeared to maintain a good appetite even when medicated with a higher dose. This finding is clinically relevant as it suggests that TOC not only prolonged survival but also helped maintain a high quality of life, which is a fundamental objective in veterinary oncology. Nevertheless, it should be acknowledged that the assessment was performed by the owner and may therefore be subject to bias, as subjective perceptions can influence scoring accuracy.

In the present case, the cat was treated with TOC at an initial dose of 2.3 mg/kg administered on a Monday–Wednesday–Friday schedule, which was increased to 3.1 mg/kg after two weeks. The cat also received meloxicam (0.1 mg/kg once daily) concurrently, and no adverse effects were observed throughout treatment, even after dose escalation. This contrasts with the findings of Harper and Blackwood (2017), who evaluated cats with various neoplastic conditions and reported a median toceranib dose of 2.78 mg/kg (range: 2.38–3.25 mg/kg), with 71% of cats experiencing some degree of toxicity, primarily mild gastrointestinal signs and myelosuppression, and two cases of severe hepatotoxicity requiring drug discontinuation [17]. Notably, cats with squamous cell carcinoma did not respond to treatment in their study [17]. Despite these toxicities, these authors observed a biological response rate of 57.1%, with a median response duration of 90 days [17]. In comparison, the absence of side effects in our case, even at a dose near the upper limit of the reported range (3.1 mg/kg versus 3.25 mg/kg), suggests individual variability in drug tolerance and highlights the importance of close monitoring during therapy.

Although other studies have reported toxicities associated with toceranib phosphate administration in cats—such as gastrointestinal signs, hematologic changes, and biochemical alterations—the drug is generally considered well tolerated [5,11,13,18]. Other authors consistently describe adverse events as predominantly mild (VCOG grade I–II), resolving with supportive care, dose adjustment, or temporary discontinuation of therapy [5,13,18]. In most cases, permanent cessation of treatment was rarely required, underscoring the manageable nature of these toxicities. In the present case, no adverse effects were observed despite dose escalation, and the decision to suspend treatment was not due to toxicity but rather a clinical choice guided by established protocols and literature recommendations, prioritizing patient welfare and quality of life.

Although feline pulmonary carcinomas are classically associated with FLDS, characterized by digital metastases, atypical metastatic patterns have been increasingly recognized [1,2,19,20]. Previous reports describe dissemination to skeletal muscle, skin, and other distant sites, highlighting the aggressive biological behaviour of these tumours [1,2]. In the present case, metastases were identified not only in a digit but also in the lung, parietal pleura, mandible, scapula, spleen, skeletal muscle, and popliteal and sublumbar lymph nodes, further supporting the adoption of the broader “MODAL syndrome” terminology proposed to encompass multi-organ and distant anatomical involvement. Although regional lymph node involvement was recently documented by Santos et al. (2023), this report appears to be the first to describe metastasis to distant lymph nodes (popliteal and sublumbar) in conjunction with such extensive multi-organ involvement [19].

A key methodological limitation, however, is that contrast-enhanced CT was not performed at the time of staging due to economic reasons, despite being the gold standard for delineating the extent of metastatic disease and for informing targeted sampling (including the primary pulmonary mass and the mandibular lesion identified at presentation). Other diagnostic procedures such as biopsy of the mandibular mass or ultrasound-guided fine needle aspirates of the lung mass were not performed, not only due to economic constraints but also because they were considered invasive, with the potential to compromise the animal’s quality of life. Consequently, ante-mortem staging findings cannot be robustly correlated with the post-mortem assessment, as detailed and standardized measurements were not acquired. These observations underscore the need for comprehensive staging and monitoring in affected cats, as metastatic spread may extend well beyond the classic digit involvement traditionally associated with FLDS.

TTF-1 is a nuclear protein expressed in human and canine type II alveolar pneumocytes as well as in bronchiolar and thyroid epithelial cells, and its diagnostic utility in feline pulmonary carcinoma, including FLDS, has been demonstrated [21,22]. Interestingly, in the present case, some cells within the papillary component of the primary tumour exhibited paranuclear staining. In human pathology, cytoplasmic immunoreactivity for TTF-1 is rarely observed and should be disregarded, with only nuclear positivity considered diagnostically relevant [23]. A recent study evaluating the immunophenotypic profile of feline pulmonary carcinomas reported an absence of TTF-1 immunoreactivity in areas of squamous differentiation [19]. In contrast, our findings revealed markedly reduced but detectable nuclear immunopositivity for TTF-1 in some squamous cells, suggesting that a subset of these cells retained expression of this transcription factor despite phenotypic divergence. It is important to note that our diagnosis of squamous differentiation was only achieved by analysis of the routine H&E stain. Despite this, the detection of TTF-1 in squamous cells may be clinically relevant, as TTF-1 expression can assist in determining the primary origin of metastatic lesions, particularly in cases with complex or atypical dissemination patterns [21].

C-kit is a receptor tyrosine kinase involved in the differentiation and survival of immature cells, and its expression is considered physiological in several cell types. However, neoplastic cells, including those of carcinomas and mast cell tumours, may also express this marker [24,25]. In canine mast cell tumours, a well-established correlation exists between c-kit expression patterns and prognosis, with diffuse immunoreactivity associated with poorer outcomes; however, similar prognostic associations have not been demonstrated in feline mast cell tumours [25,26]. In the present case, c-kit was specifically assessed because it represents one of the primary molecular targets of TOC, the tyrosine kinase inhibitor administered to this patient. Interestingly, the primary pulmonary carcinoma lacked c-kit immunoreactivity, whereas one metastatic lesion exhibited positive staining. This finding raises important considerations regarding tumour heterogeneity and therapeutic response. The absence of c-kit immunoexpression in most metastatic sites does not imply that toceranib suppressed its expression, as the drug inhibits receptor activity rather than gene expression. Instead, it suggests that only a subset of tumour cells relied on c-kit signalling, and these may have been controlled during treatment, while c-kit-negative clones continued to proliferate. Alternatively, the clinical benefit observed could have resulted from inhibition of other relevant targets, such as VEGFR2 and PDGFR, which regulate angiogenesis and tumour progression. These observations highlight the complexity of targeted therapy in feline oncology and reinforce the need for comprehensive immunohistochemical profiling to guide personalised treatment strategies. Although clinical safety of TOC in this case was observed, we could not determine if this drug was effective in the treatment of FLDS.

## 6. Conclusions

To the authors’ knowledge, this appears to be the first report describing the use of toceranib phosphate for the treatment of pulmonary carcinoma in a feline patient, specifically in a palliative setting. Remarkably, the cat presented in this case achieved a survival time that exceeded the mean values previously reported for similar conditions, suggesting a potential therapeutic benefit. Furthermore, the rapid progression of disease observed following discontinuation of toceranib strongly implies that the drug may have contributed to delaying tumour advancement during its administration, underscoring its possible role in disease control.

In this single case report, toceranib was well tolerated and allowed palliative management; however, its antitumour effectiveness cannot be determined due to key methodological limitations, including the absence of baseline contrast-enhanced CT and standardized serial re-staging. These limitations highlight the need for future prospective studies in feline pulmonary carcinoma—using rigorous staging protocols at baseline and during follow-up—to evaluate the potential therapeutic role of toceranib under controlled conditions, including its efficacy in a larger population of cats with FLDS and its possible activity across multiple receptor pathways.

## Data Availability

The original contributions presented in this study are included in the article. Further inquiries can be directed to the corresponding author.

## Abbreviations

The following abbreviations are used in this manuscript:

FLDS	Feline lung–digit syndrome
H&E	Haematoxylin and Eosin
HPF	High power-fields
MODAL	Muscle/ocular/digit/aorta/lung
PDGFR	Platelet-derived growth factor receptor
TOC	Toceranib phosphate
TTF-1	Thyroid transcription factor-1
VEGFR2	Vascular endothelial growth factor receptor 2
